# Supplementary material for: Glucose Induces Resistance to Polymyxins in High-Alcohol-Producing Klebsiella pneumoniae via Increasing Capsular Polysaccharide and Maintaining Intracellular ATP
Source: Microbiol Spectr. 2023 Jun 20;11(4):e00031-23. doi: 10.1128/spectrum.00031-23 (PMC10434286; doi:10.1128/spectrum.00031-23)
Supplement: Supplemental file 1 — Table S1. Download spectrum.00031-23-s0001.pdf, PDF file, 0.2 MB [file spectrum.00031-23-s0001.pdf]

Supplementary Table S1 Bacterial strains, plasmids and primers used in this study

Strain/ Plasmid	Description	Source/Purpose
K. pneumoniae		
W14	Wild type strain of High-alcohol-producing *K. pneumoniae* isolated from NAFLD&ABS patient	（1）
Δ*crp*	W14 deleted of *crp*	This study
Δ*crp*/ *crp*	Complemented *crp* mutant with a pGEM-T Easy plasmid	This study
Δ*crp*/Δ*manC*	W14Δ*crp* deleted of *manC*	This study
**Plasmid**		
pKO3-Km	Gene replacement plasmid derived from pKO3 with an insertion of Km resistance cassette into *AccI* site	（2）
pKO3-*crp*	pKO3 derivative, for *crp* deletion	This study
pKO3-*manC*	pKO3 derivative, for *manC* deletion	This study
pGEM-T-easy	Expression vector with an insertion of Km cassette	(Promega, Madison, WI, USA)
**Primer**	**Sequence (5′→3′)**	**Function**
KO-*crp*-upF	TCGGTACCCGGGGATCGCACTTCATAGGAGTGGGTGATG	Construction of *crp* deletion
KO-*crp*-upR	GGTTCTGATCTTCCAGCATTCATGCGCGGTTATCCTCTGTTA	
KO-*crp*-dnF	TAACAGAGGATAACCGCGCATGAATGCTGGAAGATCAGAACC	
KO-*crp*-dnR	GGTCGACTCTAGAGGATCGCAACCAGAACCAGTTAAACACG	
KO-*manC*-upF	TCGGTACCCGGGGATCGCATCGATAAACGACGCCTCG	Construction of *manC* deletion
KO-*manC*-upR	GGCCTCAGTAAATTACTGGGTGTGGGACAAAACGCAGAATGAC	
KO-*manC*-dnF	GTCATTCTGCGTTTTGTCCCACACCCAGTAATTTACTGAGGCC	
KO-*manC*-dnR	GGTCGACTCTAGAGGATCGCACGTTGATGTCATTCTTGATGAAGC	
C-*crp*-TeasyF	AATTGGGCCCGACGTCGCATGCATCTCGCACATGAACACCCTGGTGG	Expression of *crp* in HiAlc *Kpn*
C-*crp*-TeasyR	TTGGGAGCTCTCCCATATGGTCGACTAAGCGACTCCCGTAGCGGCTGTTC	
manC-RT-F	CCCCAGGGACGGTAAATC	qRT-PCR
manC-RT-R	TGTCAGCACCAAGGACGC	
wzi-RT-F	CAGTTTACCGCATCCTTACCC	qRT-PCR
wzi-RT-R	TGGAACCGACCCTTGGC	
galF-RT-F	GCCCATTTTCTTCCCGC	qRT-PCR
galF-RT-R	CTGACCGACGCCATTGC	

1. Yuan J, Chen C, Cui J, Lu J, Yan C, Wei X, Zhao X, Li N, Li S, Xue G, Cheng W, Li B, Li H, Lin W, Tian C, Zhao J, Han J, An D, Zhang Q, Wei H, Zheng M, Ma X, Li W, Chen X, Zhang Z, Zeng H, Ying S, Wu J, Yang R, Liu D. 2019. Fatty Liver Disease Caused by High-Alcohol-Producing *Klebsiella pneumoniae*. Cell Metab 30:675-688. 10.1016/j.cmet.2019.08.018.

2. Link AJ, Phillips D, Church GM. 1997. Methods for generating precise deletions and insertions in the genome of wild-type *Escherichia coli*: application to open reading frame characterization. J Bacteriol 179:6228-6237. 10.1128/jb.179.20.6228-6237.1997.
